# Treatment of Chronic Fatigue Syndrome: Findings, Principles and Strategies

**DOI:** 10.4306/pi.2008.5.4.209

**Published:** 2008-12-31

**Authors:** Patrick Luyten, Boudewijn Van Houdenhove, Chi-Un Pae, Stefan Kempke, Peter Van Wambeke

**Affiliations:** 1 Department of Psychology, University of Leuven, Leuven, Belgium.; 2 University Hospital Gasthuisberg, University of Leuven, Leuven, Belgium.; 3 Department of Psychiatry, The Catholic University of Korea College of Medicine, Seoul, Korea.; 4 Department of Psychiatry and Behavioral Sciences, Duke University Medical University, Durham, NC, USA.

**Keywords:** Chronic fatigue syndrome, Treatment

## Abstract

Chronic fatigue syndrome (CFS) is a debilitating condition characterized by serious medically unexplained mental and physical fatigue. The high prevalence and both direct and indirect health costs of CFS patients represent a huge problem for contemporary health care. Moreover, the prognosis of CFS, even when treated, is often poor. In this paper, we first critically review current evidence based treatments of CFS. Second, we discuss the growing insights into the etiopathogenesis of CFS, and the need to translate and integrate these insights into future treatments. In particular, we formulate a pragmatic and empirically testable treatment approach, tailored to the individual needs of patients, which aims at restoring the mental and physical equilibrium of CFS patients by trying to bring about sustained life style changes.

## Introduction

Chronic fatigue syndrome (CFS) is a condition characterized by serious medically unexplained mental and physical fatigue of at least 6 months duration, accompanied by sleep disturbances, poor concentration and flu-like symptoms.^1^ CFS represents a huge problem for contemporary health care because of its high prevalence (estimates vary between 0.5 to 2.5% in the general population),^2^,^3^ but also because of the associated physical and psychosocial disability, leading to high direct as well as indirect medical and societal costs. Moreover, both clinical experience^4^ and research^5^ indicate that CFS is often difficult to treat.

In this paper, we therefore first critically review current evidence based treatments of CFS. Second, we discuss the growing insights into the etiopathogenesis of CFS, and the need to translate these insights into treatment principles and strategies. Based on these considerations, we formulate a pragmatic and empirically testable treatment model that may be integrated into current evidence based treatments. In particular, this approach focuses on the tailoring of treatment for CFS patients, and aims at restoring mental and physical equilibrium in the long-run by trying to accomplish sustained changes in life style.

## Evidence Based Treatments for Chronic Fatigue Syndrome

In the absence of causative therapies, a wide variety of treatment approaches for CFS have been suggested, including pharmacological, nutritional, immunological, exercise and psychosocial treatments. However, recent meta-analyses and reviews have found that only cognitive behavioral therapy (CBT) and graded exercise treatment (GET) can be considered as evidence based treatments.^6^-^9^ In particular, on average 40-50% of CFS patients show clinical improvement in fatigue after CBT or GET compared to about 20-30% in usual care.^8^,^10^

However, many questions remain about the therapeutic aims, strategies and outcomes, as well as the purported mechanisms of change in these treatments. First, success rates of both CBT and GET leave considerable room for improvement, particularly as studies on the long-term effects of these treatments have yielded mixed conclusions.^10^ Together with the chronic and relapsing nature of CFS, these findings suggest that future studies should investigate the efficacy of maintenance treatment, which may help patients to change maladaptive core assumptions and achieve lasting life style changes.^11^ Second, concerns have been raised about the generalisability of results based on randomized, controlled clinical trials (RCTs) to routine clinical practice. Randomized trials typically include patients that are more mobile and show less psychiatric comorbidity.^10^ Moreover, patients in RCTs may be more motivated, while therapists may have higher levels of training, than in routine practice. Congruent with these assumptions, the only study that investigated the effect of CBT for CFS within versus outside the confines of an RCT found that whereas 63% of the patients in the RCT condition no longer met the fatigue caseness criterion, only 36% of patients did so in routine clinical care.^12^ Third, there is little evidence supporting the putative mechanisms of change in CBT and GET.^10^,^13^ This has led some researchers to speculate that, as for many psychological problems, patient,^14^ therapist,^5^,^15^ and common factors such as a emotional processing or the quality of the therapeutic alliance^16^ may be more important in predicting outcome than specific techniques associated with particular 'brand names' of therapy. Although further research is needed, these assumptions are congruent with findings that other treatments such as mindfulness-based treatment and counseling have been shown to lead to comparable effect sizes as CBT and GET in the treatment of CFS.^17^ Finally, psychological treatments such as CBT and GET are often not acceptable for CFS patients, and many patients therefore seek alternative treatments such as homeopathy^18^ or dubious experimental biological treatments, often through the internet.^19^

Hence, despite considerable progress in the treatment of CFS, much remains to be investigated. In the mean time, pragmatic treatment programs should be developed, taking into account not only treatment outcome studies, but also progress in research concerning the etiopathogenesis of CFS and related conditions.^20^

## Chronic Fatigue Syndrome and Stress-Related Disorders

Although the etiology of CFS largely remains elusive, there is increasing evidence that CFS belongs to the spectrum of 'Stress Intolerance and Pain Hypersensitivity' (SIPH) syndromes,^21^ characterized by a persisting inability to tolerate and recover from stress in the broad sense of the word (i.e., including physical and mental effort; sensory overload), and pathological pain processing. In particular, there is increasing evidence that the common pathophysiology of CFS and other stress-related disorders mainly consists of a dysfunction of the stress system. This dysfunction most likely involves changes in the reactivity of the hypothalamic-pituitary-adrenal (HPA) axis as well as disturbances in brain neurotransmitter balances (particularly serotonin and norepinephrine),^9^,^22^ which may be in part genetically determined.^23^,^24^ In particular, it is hypothesized that pathogenesis of these disorders might involve a HPA axis 'switch' of the from hyper- to hypofunction following a chronic or intense period of physical and/or psychosocial stress,^25^ leading to disturbed stress reactivity, and abnormal inflammatory activity which may be responsible for the 'sickness behaviour' that is often observed in these patients. More specifically, pro-inflammatory cytokines may lead to feelings of lethargia, increased fatigability, loss of concentration, generalised hyperalgesia and further hypersensitivity to stress, as well as a tendency to withdraw from the outside world.^26^

The assumption of a common pathophysiology in SIPH disorders is further supported by studies showing substantial co-occurrence among different functional somatic syndromes,^27^ such as CFS, fibromyalgia (FM) and irritable bowel syndrome^27^,^28^ as well as their overlap with affective spectrum disorders as expressed in high comorbidity and familial coaggregation with depression and anxiety.^29^-^34^

## Towards a Pragmatic Treatment Approach of Chronic Fatigue Syndrome: Principles and Strategies

The current limitations of evidence based treatments for CFS and the growing knowledge of the etiopathogenesis of CFS both emphasize the need to refine treatment approaches of CFS based on the following two principles.

The first principle entails that patients should receive a diagnostic label that subsumes their complaints within a broader set of SIPH disorders, characterized by stress intolerance and pain hypersensitivity. The advantage of such a label above the descriptive diagnosis of CFS is that it not only is in line with our current understanding of the etiopathogenesis of CFS, but also provides patients with a plausible illness theory that is not dualistic, and that also provides a plausible theory of recovery and perhaps cure. For instance, patients should be informed that their symptoms result from a loss of resilience of their stress system, most probably because of chronic burden or overload, which also has led to disturbances in their immune and pain systems. Moreover, whatever the origin of this loss of resilience, patients should be reassured that they can substantially contribute to the recovery of their stress system by finding a new equilibrium, which involves acknowledging and understanding their limitations and includes learning to better 'listen to their body'. This 'biopsychosocial' view may help patients to accept their condition and understand its multifactorial nature, but also help them work through the loss of a previous, and often overactive, life style. Finally, the idea that change will entail a long-term process of adjustment of lifestyle and life goals should be emphasized.^35^

The second principle involves the tailoring of treatment to individual patients. It has become clear by now that CFS is a heterogeneous condition.^35^,^36^ Besides optimal symptom control, including adequate treatment of psychiatric co-morbidities (e.g. antidepressants for associated depression and anxiety disorders^37^), individually tailored strategies that aim at promoting the recovery of the stress system should be integrated in current evidence based treatments or offered as complementary treatments. For instance, whereas for most CFS patients activity pacing might be helpful to prevent so-called outbursts of activity followed by post-exertional malaise, in some patients the focus must be on rebuilding physical condition, often by overcoming 'fear of movement'.^20^ In other patients, dysfunctional beliefs, such as catastrophizing, must be addressed. More directive treatments such as CBT^14^ or mindfulness and acceptance based strategies^38^ might work best for some patients. Other patients, however, might need less directive treatments, such as counselling,^17^,^39^ psychodynamic treatment,^40^,^41^ or systemic interventions^42^ to address psychiatric comorbidity, personality and/or relational problems, and to adjust life goals and work through the "loss of a life style". Moreover, such tailored treatments best occur based on a coherent treatment plan within the context of a multidisciplinary approach^39^ that also addresses medical-social issues, as these may considerably influence the treatment process.^14^

## Conclusions

Notwithstanding considerable advances in the treatment of CFS, current evidence based treatments clearly provide no panacea. In particular, more research is needed concerning the long-term effects of these treatments, their generalisability to routine clinical care, and the identification of their mechanisms of change. In the meantime, a pragmatic treatment approach is proposed that can be integrated in current evidence based treatments of CFS. This treatment approach emphasizes the need to incorporate findings concerning the etiopathogenesis of CFS into treatment, as well as tailoring treatment to individual patients.

This approach, it is believed, will help find patients a new mental and physical equilibrium by adjusting their life style and life goals. Further research, however, is needed to test these assumptions, and particularly to find out "what works for whom".^43^ Yet, it is likely that in the near future, new treatments that are based on our increasing insights into the etiopathogenesis of CFS will emerge.
